# Investigation of Factors Affecting Aerodynamic Performance of Nebulized Nanoemulsion

**Published:** 2016

**Authors:** Hosein Kamali, Shayan Abbasi, Mohammad Ali Amini, Amir Amani

**Affiliations:** a *Targeted Drug Delivery Research Center, Mashhad University of Medical Sciences, Mashhad, Iran.*; b *Research Center of Natural Product Safety and Medicinal Plants, North Khorasan University of Medical Sciences, Bojnurd , Iran. *; c *Institute of Biochemistry and Biophysics (I.B.B), University of Tehran, Tehran, Iran. *; d *Department of Microbiology, Faculty of Veterinary Medicine, Islamic Azad University, Karaj branch, Karaj, Iran .*; e *Department of Medical Nanotechnology, School of Advanced Technologies in Medicine, Tehran University of Medical Sciences, Tehran, Iran. *; f *Medical Biomaterials Research Center, Tehran University of Medical Sciences, Tehran, Iran.*

**Keywords:** Nanoemulsion, Micelle, Budesonide, Nebulizer

## Abstract

This work aimed to prepare a nanoemulsion preparation containing budesonide and assess its aerodynamic behavior in comparison with suspension of budesonide. *In-vitro* aerodynamic performance of the corresponding micellar solution (ie. nanoemulsion preparation without oil) was investigated too. Nanoemulsions of almond oil containing budesonide, as a hydrophobic model drug molecule, were prepared and optimized. Then, the effect of variation of surfactant/co-surfactant concentration on the aerodynamic properties of the nebulized aerosol was studied. The results indicated that the most physically stable formulation makes the smallest aerodynamic size. The concentration of co-surfactant was also shown to be critical in determination of aerodynamic size. Furthermore, the optimized sample, with 3% w/w almond oil, 20% w/w Tween 80+Span 80 and 2% w/w ethanol showed a smaller MMAD in comparison with the commercially available suspension and the micellar solution.

## Introduction

Nanoemulsions, submicron sized and transparent/translucent emulsions, have been defined as colloidal dispersions of two immiscible phases (ie. an oil and an aqueous phase) which are stabilized by surfactant and co-surfactant (1, 2). Spontaneous formation of nanoemulsions is a result of low interfacial tension which leads to thermodynamically and/or kinetically stable dispersions (1).

 In addition to the ease of preparation and solution-like properties of nano emulsions, they offer a wide range of advantages in drug delivery purposes, including the ability to load different bioactive agents (3) with both hydrophilic and lipophilic drug molecules, protection of active ingredient(s) from hydrolysis and oxidation as well as enhancing bioavailability and rate of absorption (4, 5). This has made them a center of attention for pharmaceutical industries and academia to study their applications in cosmetics (5), antimicrobial agents, mucosal vaccines (6), cell cultures (1) and various drug delivery systems, including inhalation delivery systems (7, 8).

In our previous works, for the first time, we compared a nanoemulsion system with the commercially available microsuspension of budesonide. The nanoemulsion contained a medium chain triglyceride (MCT) oil, polysorbate 80 and ethanol. The aerodynamic behavior of the preparation was investigated in two different types of nebulizers. A significant improvement for *in-vitro* performance of the aerosolized nanoemulsion was shown compared with the suspension, which was probably due to smaller hydrodynamic sizes (9). In another study, a preparation with hydrodynamic particle size of around one-third the size of commercial suspension, showed a slightly smaller aerodynamic size (10). This could be a confirmation to the above mentioned hypothesis, saying that smaller particle size can lead to better aerodynamic behavior. A second mechanism which could contribute to production of smaller aerosol droplets is higher concentration of surfactant in the nanoemulsion which makes the interfacial tension smaller, thus, leads to generation of smaller aerosol droplets. To the best of our knowledge, no other work has yet considered mechanism(s) underlying the superior performance of nanoemulsions. The aim of this work is to examine the aerodynamic behavior of several nanoemulsion systems, different from our previous one. Subsequently, the effect of surfactant/co-surfactant concentrations on aerodynamic behavior of the nebulized nanoemulsion will be studied. The optimized preparation will then be compared with the micellar solution (ie. nanoemulsion without oil) and the commercially available microsuspension of budesonide. This would shed a light on factors affecting the aerodynamic behavior of a nanoemulsion.

## Materials and methods

*Materials*

Sweet almond oil, Tween 80, Span 80 and alcohol were from Sigma-Aldrich (Germany). The budesonide was from Industriale Chimica s.r.l. (Italy).

*Preparation of nanoemulsion*

The nanoemulsion formulation was consisted of Span 80 and Tween 80 (i.e. surfactant system), ethanol (i.e. co-surfactant), and almond oil (i.e. inner phase) in de-ionized water. In our previous work, it was shown that the most physically stable preparation of this nanoemulsion, contained 20% (w/w), 2% (w/w) and 3% (w/w) of surfactant, co-surfactant and oil, respectively (ie. S1) (11). To examine the effect of variation of the ingredients concentration on the aerodynamic behavior, surfactant concentration was modified to 15% (w/w) (ie. S2) and 25% (w/w) (ie. S3). Similarly, the co-surfactant concentration was increased to 4% (w/w) (ie. S4) and 8% (w/w) (ie. S5) to investigate its effect on the aerodynamic diameter. Samples with co-surfactant concentrations less than 2% (w/w) did not show acceptable stability for nebulization studies, thus, were excluded from aerosolization experiments. Additionally, a micellar solution was prepared by elimination of oil from S1 (ie. 20% (w/w) surfactant and 2% (w/w) co-surfactant). The hydrodynamic and aerodynamic properties of the solution were then compared with the S1. Budesonide was added to all preparations in final concentration of 250 µg/mL (i.e. similar to concentration of budesonide in commercial suspension, Pulmicort Respules® (Astrazeneca, UK)).

*Particle size measurement*

Photon correlation spectroscopy (PCS) technique using Zetasizer® NanoZS (Malvern Instruments, UK) was used to determine the average particle size diameter (Z-Average) and the polydispersity index (PDI). Dispersions were analyzed without dilution. Accuracy of the instrument was calibrated by a Nanosphere^TM^ size standard, 500 nm (Duke Scientific Corporation, USA).

**Figure 1:**
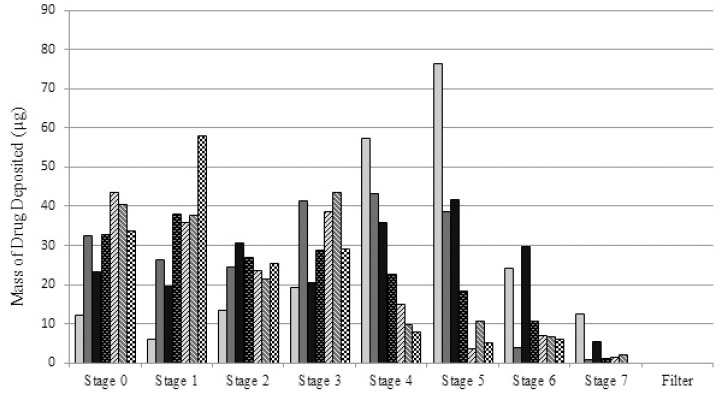
*In-vitro *deposition profile of nanoemulsion, micellar and suspension samples using Anderson cascade impactor

**Table 1:** The composition of samples understudy and their obtained hydrodynamic particle size

	Water % (w/w)	Oil % (w/w)	Tween + Span % (w/w)	Co-surfactant % (w/w)	Particle Size (nm)	PDI
S1	75.0	3.0	20.0	2.0	45.0	0.30
S2	80.0	3.0	15.0	2.0	50.9	0.35
S3	70.0	3.0	25.0	2.0	73.0	0.20
S4	73.0	3.0	20.0	4.0	49.3	0.26
S5	69.0	3.0	20.0	8.0	105.9	0.14
Micelle	78.0	0.0	20.0	2.0	55.6	0.34

**Table 2:** Mean (SD) for aerodynamic characterization results of nanoemulsion samples (n = 3).

	MMAD (SD) (µm)	GSD (SD)	FPF (%)
S1	2.07 (0.45)	1.91 (0.31)	84.3 (11.0)
S2	3.86 (0.60)	2.02 (0.28)	65.7 (8.5)
S3	2.93 (0.56)	2.51 (0.24)	64.1 (10.2)
S4	5.01 (0.91)	1.74 (0.15)	44.0 (6.8)
S5	5.53 (1.08)	1.81 (0.26)	37.9 (5.8)

**Table 3.** Mean (SD) for aerodynamic characterization results of micellar solution and commercial suspension

	MMAD (SD) (µm)	GSD (SD)	FPF (%)
Micelle	5.36 (0.98)	1.95 (0.28)	40.0 (5.7)
Commercial suspension	6.17 (1.2)	1.57 (0.25)	29.1 (4.4)
S1	2.07 (0.45)	1.91 (0.31)	84.3 (11.0)

*In-vitro aerosol characterization*

To determine the aerodynamic particle size of each formulation, samples (5 mL) were nebulized by a jet nebulizer (Beurer-Medical jet nebulizer IH25, Ulm, Germany) at ambient temperature and flow rate of 28.3 L/min into an Anderson 8-stage cascade impactor equipped with filters on each stage. The mouthpiece of the nebulizer was directly connected to the cascade induction port. The set up was kept running till no aerosol was visually detected leaving the nebulizer. Further details about the setup have been given previously (12). The amount of budesonide deposited on each stage of the cascade impactor and the final filter was determined by HPLC, as reported earlier (9, 13).

Mass median aerodynamic diameter (MMAD) and geometric standard deviation (GSD) values were calculated using Online MMAD Calculator (14). Fine particle fraction (FPF) was calculated using the following equation: 

$\mathrm{FPF}=\frac{\mathrm{amount of particles}<5\mu m}{\mathrm{emitted dose}}$

*HPLC analysis*

HPLC system was equipped with a solvent delivery system (KnauerSmartline, Ulm, Germany) connected with a pump (Smartline Pump 1000), a ChromSep HPLC column C18 reverse-phase (10 m, 250 mm × 4.6 mm i.d.) (Varian Cromopack), a UV-Vis detector (Smartline PDA Detector 2800) and interfaced to ChromGate® Stand-Alonesoftware. Mobile phase consisted of acetonitrile/ phosphate buffer having 0.025 M of KH_2_PO_4_ and Na_2_HPO_4_ each (45: 55) and pH 3.5 adjusted with phosphoric acid and was delivered at a flow rate of 1 mL/min. Injected volume for each sample, dissolved in methanol, was 30 µL. The UV detector was set at 240 nm and retention time of budesonide was determined to be ~7 min. All the experiments were performed three times and at ambient temperature.

## Results and discussion

*Hydrodynamics particle size*

In our previous work (9), utilizing the solution-like properties of nanoemulsion, we investigated a nanoemulsion preparation to overcome several disadvantages of nebulization of microsuspensions (15). To continue our work, here in, we studied a similar nanoemulsion preparation. At the first stage, we aimed to investigate the effect of variation of ingredients concentration from its most stable condition on hydrodynamic and aerodynamic size. We then compared the optimum preparation with the commercial suspension of budesonide and the micellar solution.

The results of hydrodynamic particle size, expressed as mean Z-average size of three replicates, determined at 25 °C, in addition to polydispersity index (PDI), obtained from PCS, are given in Table 1. PCS analysis of budesonide nanoemulsion showed a minimum particle size for S1 (45 nm).

From Table 1. S1, the most stable preparation (11), containing 20% (w/w) surfactant mixture (Tween 80: Span 80 (57:43)), 2% (w/w) ethanol and 3% (w/w) almond oil, shows the smallest particle size with appropriate PDI value. Variation of surfactant concentration from its optimum value increased the particle size. The effect of surfactant in stabilizing the nanoemulsion particles, both in the solution and within the aerosol droplets, is related to its ability to cover the surface of particles due to steric effects (i.e. when a molecule/group with a large size prevents physical/chemical reactions with other molecules/groups (16)). Decreasing the particle size with increasing the surfactant concentration up to an optimum value has been well documented in the literature (eg. (17)). However, depending on other independent variables, this effect has been reported not to be linear. Even in some cases, an opposite effect has been reported (18, 19). The change in the hydrodynamic particle size as a function of surfactant concentration (ie. S3 *vs.* S1) is possibly due to migration of the excess surfactant molecules to the surface of the particles which makes particles larger as detailed elsewhere (11, 20). Some other mechanisms which have been suggested to increase the observed particles size are Ostwald ripening due to enhance in Brownian motion, increase in the number of collisions between the particles due to increase in number of particles and formation of lamellar liquid crystalline structures (11, 20). Similar mechanisms may be involved in enhancing the size when co-surfactant concentration increases (ie. S4 & S5 *vs.* S1). Additionally, it is already shown that co-surfactant molecules act as a second amphiphil and locate at the interfacial film (7). Nevertheless, at higher amounts of co-surfactant, the co-surfactant molecules may replace the surfactant molecules, therefore, make the nanoemulsion droplets less stable. This, also can occur due to the hydrogen binding between alcohol and surfactant molecules, making the interfacial film less fluid (21). Besides, alcohol molecules can penetrate to the inner phase which causes an increase in size, thus, destabilize the system by expanding the interfacial film (22). All these phenomena contribute to enhancing the size of generated nanoemulsion particles.

The results of PDI studies show that increase in concentration of either surfactant or co-surfactant causes a decrease in PDI (ie. S3, S4 and S5 *vs.* S1). Considering the size results whereby the particle size of the samples has become larger, it could be argued that the excess surfactant/co-surfactant molecules have “gathered” other smaller particles to make a homogenous preparation with nanoemulsion particles lesser in number but larger in size.

*Aerodynamic particle size*

*Determination of factors affecting the aerodynamic particle size in nanoemulsions*

Mean (SD) aerodynamic particle size data is provided in Table 2. As the details show, in line with the hydrodynamic particle sizes, a change in either of surfactant or co-surfactant concentration from the optimum point makes a significant increase (p<0.05) in MMAD. The FPF value for the S1 is also larger than all other samples. This could be due to the smallest hydrodynamic particles size obtained for the nanoemulsion particles. We believe that stability of the nanoemulsion droplets which makes them remain intact during nebulization process may also be a reason for small aerosol droplets. Notably, the increase in the concentration of surfactant/co-surfactant is not decreasing the aerosol droplets size. Additionally, despite a relatively large variation in PDI values, the differences in GSD values are not significant, showing that PDI may not be a dominant factor in determining the deviation in size of aerosol droplets.

Furthermore, by comparing the data of S1 with those of S4 and S5, a considerable increase in MMAD of S4 and S5 is observed. In other words, the difference in aerodynamic behavior becomes more prominent, when the concentration of co-surfactant changes. Such a considerable increase shows that the co-surfactant plays an important role in stabilizing the aerosol droplets, regardless of hydrodynamic particle sizes.

Comparing the FPF values in Table 2. a significant change (p<0.05) in FPF values is observed when co-surfactant concentration is changing (84.3, 44.0 and 37.9 for S1, S4 and S5, respectively). This change is not significant in case of variation of surfactant concentration, though. This confirms the importance of co-surfactant in generating small aerosol droplets. It is arguable that increase in the co-surfactant concentration from its optimum value makes the emulsion less stable (as mentioned earlier), leading to larger hydrodynamic and aerodynamic diameters. Considering the fact that smaller particles require less energy to be aerosolized and incorporated in aerosol droplets, the importance of smaller particle sizes to provide smaller aerosol particles may be recognized. In total, it may be concluded that the most stable nanoemulsion preparation has the optimum *in-vitro* aerodynamic behavior. This sample was then selected for the comparison studies with micellar solution and commercial suspension.

*Comparison of the aerodynamic particle size of the nanoemulsion preparation with that of commercial suspension and micellar solution*

Table 3. summarizes the aerodynamic results obtained for the micelle/suspension preparations. The results show a substantial increase in the MMAD of micelle and commercial suspension compared with the nanoemulsion preparation, respectively. Similar pattern is also observed for the FPF of the preparations. Taking into account the smaller particles and smaller aerosol droplets, an improved drug absorption rate (23) and *in-vivo* efficacy (24) is also anticipated for the nanoemulsion.

Comparing the hydrodynamic sizes of the micellar solution with that of nanoemulsion reveals that presence of oil is probably associated with providing proper intra-particle interactions to tighten the structure and making smaller particles. This will also lead to a type of resistance against the shear forces applied by nebulizer to aerosolize the solution. In general, it is arguable that worsening the aerodynamic behavior of the samples, when deviating from optimum oil/surfactant/co-surfactant concentration, is most probably due to instabilities occurred in the preparation. The mechanisms proposed above for increasing the hydrodynamic particle size, could be reasons for the increase in the aerodynamic particle size too.

To obtain a clinically acceptable preparation for nebulization purposes, apart from satisfactory performance findings, the preparation should be safe for human uses. For inhalation purposes, it is worth noting that the majority of nanoemulsions reported in the literature have employed surfactants/co-surfactants that have not been approved by FDA, yet (25, 26). Moreover, when considering inhalation, a limited number of safety reports may be found in the literature for most of surfactants/co-surfactants as well as their corresponding concentrations. Therefore, safety profiles of such preparations should be considered too. 

## Conclusion

This study aimed to continue the work on the use of nanoemulsions in nebulizers. It was shown that the most stable preparation with the smallest hydrodynamic particle size (as obtained from PCS), has the best *in-vitro *aerodynamic behavior. In total, variation of surfactant/co-surfactant led to less *in-vitro *aerodynamic performance. Furthermore, the nanoemulsions showed a better *in-vitro *efficacy compared to micelle and commercial suspension. 
